# Clinical analysis and comparative study of facial symmetry of three-dimensional skull models

**DOI:** 10.1371/journal.pone.0329322

**Published:** 2025-07-31

**Authors:** Zhengrui Zhang, Aijia Deng, Hao Li, Yunming Li, Jianhua Wei, Yan Wang

**Affiliations:** 1 Department of Stomatology, The General Hospital of Western Theater Command, Chengdu, Sichuan Province, China; 2 State Key Laboratory of Oral & Maxillofacial Reconstruction and Regeneration, National Clinical Research Center for Oral Diseases, Shaanxi Key Laboratory of Stomatology, Department of Oral and maxillofacial head and neck Cancer surgery, School of Stomatology, The Fourth Military Medical University, Xi’an, China; 3 Department of Stomatology, Chongqing Western Hospital, Chongqing, China; 4 School of Stomatology of Southwest Medical University, Luzhou, Sichuan Province, China; 5 Department of Information, Medical Support Center, The General Hospital of Western Theater Command, Chengdu, Sichuan Province, China; Universiti Sains Malaysia, MALAYSIA

## Abstract

Facial symmetry is a critical determinant in maxillofacial reconstruction. To establish an ideal midsagittal plane (MSP) for three-dimensional (3D) skull model in the diagnosis and treatment of maxillofacial reconstructing and unilateral maxillofacial lesions, the maxillofacial spiral computed tomography data from 51 patients with normal craniofacial anatomy in the Department of Stomatology, The General Hospital of Western Theater Command, Chengdu, Sichuan Province, China, were collected to performed 3D reconstruction. Every 3D skull model established three common MSPs: N-ANS-PNS, N-ANS-S, and N-Ba-S by corresponding anatomical landmarks. The original 3D skull models were mirrored using different MSPs to construct mirror models. The MSPs accuracy was assessed through repeated measures analysis combined with the 3D chromatic deviation mapping. Quantitative comparisons revealed statistically significant differences (*P* < 0.05) in overlap precision across MSP definitions. Mean deviation values (± SD) between the mirror and original models of N-ANS-PNS, N-ANS-S, and N-Ba-S were −1.1415 ± 0.6651, −0.9075 ± 0.6279, and −0.3961 ± 0.7970 mm respectively. Gender-based analysis demonstrated significantly better facial symmetry in female models compared to males across all MSP definitions (*P* < 0.05), while demographic factors (age, height, and weight) showed no statistically significant correlation with symmetry outcomes (*P* > 0.05). These findings validate the efficacy of mirroring technology combined with 3D chromatic deviation mapping for MSP accuracy assessment. The N-ANS-PNS plane emerged as the most reliable reference for facial symmetry evaluation in 3D skull models, with female morphology exhibiting inherently superior bilateral symmetry compared to male counterparts.

## 1. Introduction

Facial symmetry is one of the important indicators to measure human facial aesthetics [1–3]. This symmetry is also one of the basic goals of surgeons for aesthetic repair and facial reconstruction [4–6]. With the popularization of three-dimensional (3D) technology, the establishment of a midsagittal plane (MSP) has become increasingly important. Many studies have proved that creation of the 3D mirror model based on the standard MSP can restore and reconstruct the normal shape of the target area to a great extent; for example, based on the mirror reconstruction model, it is possible to establish the surgical resection range of fibrous hyperplasia of the jaw and to realign the unilateral maxillofacial fracture segment [7–9]. Accurate MSP can divide the head and maxillofacial into two completely symmetrical parts. The mirror simulation operation of the 3D skull model with the MSP provides an important reference for the surgical procedure to restore the original shape of the maxillofacial area [10,11]. However, multiple factors affect human maxillofacial development, such as biological factors and environmental factors [12,13], and research has shown that the development of facial mismatch is unpredictable [14–16]. In addition, in theory, no complete symmetry exists, which results in no recognized standard process or specific method to establish MSP.

At present, establishing MSP by cranial anatomic landmarks is the most common method in clinical applications [17–19]. This approach is based on a variety of stereoscopic 3D imaging methods, such as facial images collected by laser scanners, 3D reconstructed images, or computed tomography (CT) images [20–24]. However, the method of establishing MSP is currently controversial. Studies have proposed a number of cranial anatomic points for positioning the ideal MSP [25–27], but the statistical data are insufficient to propose a standard MSP for clinical use. This study compared three common MSPs [25–27]. The CT data for 51 patients with a normal jaw were used to reconstruct the 3D skull model, with the corresponding MSP as the symmetry plane, and the overlap precision of the mirror model and original model was evaluated to select the ideal maxillofacial MSP. This study will help surgeon more effectively and reliably complete clinical treatment in maxillofacial surgery such as bone reconstruction and orthognathic surgery.

## 2. Materials and methods

Maxillofacial spiral CT data were collected for 51 patients with a normal jaw in the Department of Stomatology of the General Hospital of Western Theater Command from 28/06/2018–09/10/2021. Inclusion criteria were no craniofacial malformations, no history of maxillofacial trauma or surgical treatment, and clear imaging data on spiral CT. The study was approved by the Medical Ethics Committee of the General Hospital of Western Theater of Operations and a written certificate was obtained (Approval No. 2021EC5-121). The enrolled participants signed an informed consent form. The authors had access to information that could identify individual participants during and after data collection.

### 2.1. Methods

#### 2.1.1. Construction of a 3D skull model.

The 64-row maxillofacial spiral CT image data for 51 patients were saved in DICOM format files and imported into Mimics Research 21.0 (Materialize, Leuven, Belgium). The data were accessed for research purposes from 01/02/2022–01/06/2022. Appropriate threshold ranges (minimum 400 Hu; maximum 3071 Hu) were used to establish the jaw bone mask to reconstruct a 3D skull model.

#### 2.1.2. Establishment of the MSP.

The first step was to locate the anatomic landmark points to establish three MSPs according to the sagittal plane, coronal plane, and cross-section of the 3D skull model. Every object took three MSPs as follow and the same MSPs belonged to the same group (three groups in total):

1) *Plane N-ANS-PNS* comprised the nasal root point, anterior nasal spine point, and posterior nasal spine point.
2) *Plane N-ANS-S* comprised the nasal root point, anterior nasal spine point, and sella point.
3) *Plane N-Ba-S* comprised the nasal root point, skull base point, and sella point.

**Fig 1:**
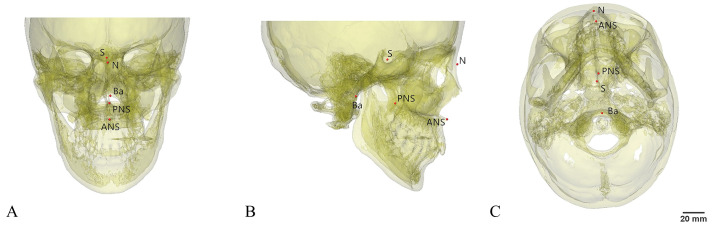
Location of anatomic landmarks on the three-dimensional skull model.

The positioning of all anatomic landmarks was completed twice by a senior physician, and the midpoint was chosen as final marker (Fig 1).

#### 2.1.3. Generation of the mirror model.

**Fig 2:**
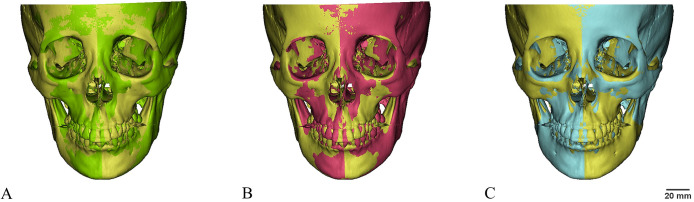
Mirror model matched to the original model (yellow). A. Green: mirror model with N-ANS-PNS as the symmetry plane; B. Red: mirror model with N-ANS-S as the symmetry plane; C. Blue: mirror model with N-Ba-S as the symmetry plane.

Using the Mimics Research 21.0 software, the steps were as follows: 1) execute “3D TOOLS-Mirror”; 2) choose the original model as the target and choose the three planes of N-ANS-PNS, N-ANS-S, and N-Ba-S as the mirrors plane; and 3) click “OK” to automatically generate three mirror models (Fig 2).

#### 2.1.4. Creation of a comparative analysis.

**Fig 3:**
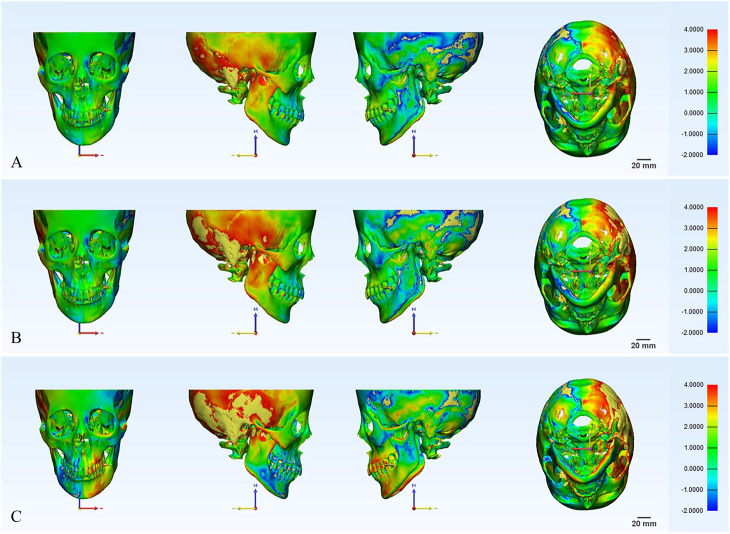
Original model and the mirror model in the heat map analysis of the 3-Matic software. A. Comparative analysis between the original model and the mirror model with the N-ANS-PNS plane as the symmetry plane; B. Comparative analysis between the original model and the mirror model with the N-ANS-S plane as the symmetry plane; C. Comparative analysis between the original model and the mirror model with the N-Ba-S plane as the symmetry plane.

The original 3D skull model and the mirror model were imported into 3-Matic Research 13.0 (Materialize) to create a comparative analysis in the STL file format. After importing, the next steps were to set the original 3D skull model to “Entity” and the mirror model to “Target Entity”; next, the command “Analyze-Create a Part Comparison Analysis.” was executed. The overlap precision between the original model and the mirror model was compared for each point, and the average overlap precision and standard deviation of the two different models were automatically calculated. The different colors showed the difference in the overlap precision at different positions. The green area represented the overlap between the mirror model and the original model, that is, the closer the value was to 0, the higher the degree of matching between the two models. The red area represented the recessed part (the mirror model surface was recessed on the surface of the original model), and the blue area represented the protruding part (the mirror model surface protruded from the meta model surface). The larger the value, the darker the color, and the greater the difference and the lower the degree of matching between the original model and the mirror model (Fig 3).

#### 2.1.5. Statistical analysis.

The values of the overlap precision between the original model and the mirror model were collected for 51 patients. Their sex, age, height, and weight and body mass index data were studied as confounding factors. The three sets of data were imported into SPSS 25.0 (SPSS, Chicago, IL, USA), and repeated ANOVA was performed. A values of *P* < 0.05 was considered statistically significant.

## 3. Results

The sex distribution was 25 male and 26 female patients. Their average values for the confounding factors were age 43.04 ± 14.27 years, height 163.96 ± 8.09 cm, weight 64.93 ± 12.09 kg, and body mass index 24.0805 ± 3.6207 kg/m^2^ (Table 1). To perform repeated ANOVA, the Shapiro–Wilk test (*P* > 0.05) showed that the values of the overlap precision were normally distributed after natural logarithmic transformation (Table 2). Preliminary modeling showed that age, height, and weight had no effect on the experimental results, whereas sex and groups (N-ANS-PNS, N-ANS-S, and N-Ba-S) had a significant effect on the results.

**Table 1:** Patient characteristics by sex.

Characteristic	Sex	Mean	Standard deviation	N
Age (years)	Male	41.76	12.791	25
	Female	44.27	15.751	26
	Total	43.04	14.273	51
Height (mm)	Male	169.12	7.541	25
	Female	159.00	4.942	26
	Total	163.96	8.094	51
Weight (kg)	Male	70.480	11.8079	25
	Female	59.596	9.9015	26
	Total	64.931	12.0888	51
Body mass index	Male	24.5634	3.21603	25
	Female	23.6162	3.97891	26
	Total	24.0805	3.62065	51

**Table 2:** Tests of normality.

MSP Model	Kolmogorov–Smirnov^a^			Shapiro–Wilk		
	Statistic	df	*P* value	Statistic	df	*P* value
N-ANS-PNS	0.128	51	0.037	0.968	51	0.184^**^
N-ANS-S	0.082	51	0.200^*^	0.971	51	0.249^**^
N-Ba-S	0.057	51	0.200*	0.985	51	0.768^**^

*Lower bound of the true significance.

**Shapiro–Wilk test: *P* > 0.05 showed that the values of the overlap precision were normally distributed after natural logarithmic transformation.

^a^ Lilliefors significance correction.

*Plane N-ANS-PNS* comprised the nasal root point, anterior nasal spine point, and posterior nasal spine point.

*Plane N-ANS-S* comprised the nasal root point, anterior nasal spine point, and sella point.

*Plane N-Ba-S* comprised the nasal root point, skull base point, and sella point.

Repeated measures analysis was performed with group as within-subjects variables and sex as between-subjects factors. The values of the overlap precision did not fit the Mauchly test of sphericity (*P* < 0.05) (Table 3). After Greenhouse–Geisser, Huynh–Feldt and lower bound corrections, the tests of within-subjects effects indicated that the values of different groups were statistically different (*P* < 0.05) (Table 4), namely, different MSPs had different effects on evaluating facial symmetry, and there was no interaction between sex and group (*P* > 0.05) (Table 4). The tests of within-subjects contrasts showed that the different MSPs had the same difference in assessing facial symmetry for different sex (*P* > 0.05) (Table 5). After controlling for gender confounders, in the male group, the three different MSPs showed a statistical difference, and the trend of that difference was the same as in the female group. However, the tests of between-subjects effects had shown that the same MSP assessed facial symmetry better in women than in men (*P* < 0.05) (Table 6).

**Table 3:** Mauchly test of sphericity^a^.

Measure: Overlap precision							
Within-Subjects Effect	Mauchly W	Approximate Chi-Square	df	*P* value	Epsilon^b^		
					Greenhouse–Geisser	Huynh–Feldt	Lower bound
Group	0.721	15.734	2	<0.001^*^	0.782	0.819	0.500

Tests the null hypothesis that the error covariance matrix of the orthonormalized transformed dependent variables is proportional to an identity matrix.

* *P* < 0.05: The values of the overlap precision did not fit the Mauchly test of sphericity.

^a^ Design: Intercept + Sex

Within-Subjects Design: Group.

^b^May be used to adjust the degrees of freedom for the averaged tests of significance. Corrected tests are displayed in the Tests of Within-Subjects Effects table (Table 4).

**Table 4:** Tests of within-subjects effects.

Measure: Overlap precision						
Source		Type III Sum of Squares	df	Mean Square	F	*P* value
Group	Sphericity assumed	14.821	2	7.410	28.772	<0.001^*^
	Greenhouse–Geisser	14.821	1.563	9.482	28.772	<0.001^*^
	Huynh–Feldt	14.821	1.638	9.046	28.772	<0.001^*^
	Lower bound	14.821	1.000	14.821	28.772	<0.001^*^
Group * sex	Sphericity assumed	0.125	2	0.062	0.243	0.785^**^
	Greenhouse–Geisser	0.125	1.563	0.080	0.243	0.730^**^
	Huynh–Feldt	0.125	1.638	0.076	0.243	0.741^**^
	Lower bound	0.125	1.000	0.125	0.243	0.625^**^
Error (Group)	Sphericity Assumed	25.241	98	0.258		
	Greenhouse–Geisser	25.241	76.593	0.330		
	Huynh–Feldt	25.241	80.281	0.314		
	Lower bound	25.241	49.000	0.515		

*P < 0.05: Values of different groups were statistically different.

**P > 0.05: No interaction between sex and group.

**Table 5:** Tests of within-subjects contrasts.

Measure: Overlap precision						
Source	Group	Type III Sum of Squares	df	Mean Square	F	*P* value
Group	Linear	14.177	1	14.177	36.271	<0.001
	Quadratic	0.644	1	0.644	5.181	0.027
Group ^*^ sex	Linear	0.010	1	0.010	0.025	0.876
	Quadratic	0.115	1	0.115	0.928	0.340*
Error (Group)	Linear	19.153	49	0.391		
	Quadratic	6.088	49	0.124		

* *P* > 0.05: Different MSPs had the same difference in assessing facial symmetry for different sex.

**Table 6:** Tests of between-subjects effects.

Measure: Overlap precision					
Transformed Variable: Average					
Source	Type III Sum of Squares	df	Mean Square	F	*P* value
Intercept	100.593	1	100.593	118.034	<0.001
Sex	6.470	1	6.470	7.592	0.008^*^
Error	41.759	49	0.852		

*P < 0.05: The same MSP assessed facial symmetry better in women than in men.

The overlap precision of group N-ANS-PNS, N-ANS-S, and N-Ba-S were −1.1415 ± 0.6651 mm, −0.9075 ± 0.6279 mm, and −0.3961 ± 0.7970 mm, respectively (Table 7). The post hoc multiple comparisons for groups indicated that the differences in facial symmetry assessed by different MSPs were statistically significant (*P* < 0.05) (Table 8): N-Ba-S was the best method, N-ANS-S was the next-best method, and N-ANS-PNS was the least preferred method.

**Table 7:** Descriptive statistics of overlap precision.

Group	Sex	Mean	Std. Deviation	N
N-ANS-PNS	Male	−0.9615	0.50339	25
	Female	−1.3146	0.76018	26
	Total	−1.1415	0.66510	51
N-ANS-S	Male	−0.6582	0.50184	25
	Female	−1.1473	0.65185	26
	Total	−0.9075	0.62793	51
N-Ba-S	Male	−0.1963	0.86490	25
	Female	−0.5882	0.68851	26
	Total	−0.3961	0.79703	51

*Plane N-ANS-PNS* comprised the nasal root point, anterior nasal spine point, and posterior nasal spine point.

*Plane N-ANS-S* comprised the nasal root point, anterior nasal spine point, and sella point.

*Plane N-Ba-S* comprised the nasal root point, skull base point, and sella point.

**Table 8:** Pairwise comparisons for groups.

(I) Group	(J) Group	Mean Difference (I–J)	Std. Error	*P* value ^a^	95% Confidence Interval for Difference ^a^	
					Lower Bound	Upper Bound
N-ANS-PNS	N-ANS-S	−0.235^*^	0.092	0.041	−0.463	−0.008
	N-Ba-S	−0.746^*^	0.124	0.000	−1.053	−0.439
N-ANS-S	N-ANS-PNS	0.235^*^	0.092	0.041	0.008	0.463
	N-Ba-S	−0.511^*^	0.081	0.000	−0.711	−0.310
N-Ba-S	N-ANS-PNS	0.746^*^	0.124	0.000	0.439	1.053
	N-ANS-S	0.511^*^	0.081	0.000	0.310	0.711

Based on estimated marginal means.

*The mean difference is significant at the.05 level.

^a^ Adjustment for multiple comparisons: Bonferroni.

*Plane N-ANS-PNS* comprised the nasal root point, anterior nasal spine point, and posterior nasal spine point.

*Plane N-ANS-S* comprised the nasal root point, anterior nasal spine point, and sella point.

*Plane N-Ba-S* comprised the nasal root point, skull base point, and sella point.

## 4. Discussion

Facial symmetry is one of the important benchmarks for evaluating facial aesthetics [28–30]. In the past, surgeons usually estimated the symmetry of the patient’s face based on their own experience to restore the facial contour during surgery, which often caused differences in postoperative results from the patient’s expectations. Therefore, preoperative accurate assessment of the patient’s facial condition, prediction of postoperative changes in facial contours, and precise surgical planning all play a vital role in the process of maxillofacial reconstruction [10,11,31,32]. The evaluation of facial symmetry usually requires specific measurement methods which are based mainly on anatomical landmarks, original-mirror alignment, and deep learning algorithms [33]. The primary common methods include direct anthropometric measurements, traditional two-dimensional (2D) cephalometric measurements, 3D photogrammetry, 3D ChromoScan and CT. According to the 3D photogrammetry research, Taylor et al. [20] found that the largest asymmetry area of the face existing in the upper one-third of the face accounts for about 10% of asymmetry, the middle one-third of the face for 49%, and the lower one-third of the face for 41%. Some scholars also posited that the main cause of facial asymmetry is bilateral asymmetry of the mandible. Kwon et al. [34] evaluated facial symmetry through mirroring technology with 3D imaging. They found that the evaluation of maxillofacial asymmetry through multiple symmetrical landmarks in 2D or 3D images could only be restricted to a relatively limited area; however, it was impossible to evaluate the difference in the overall shape of the 3D model. In contrast, the mirroring technology directly reflected the overlapping and non-overlapping areas of two 3D models in an intuitive and visual way, and it reflected the similarity index of two 3D models through the image color and multiple comparison parameters. For the mirroring technology, the precise symmetry plane is an important quantitative tool that is essential for the clinical application, such as patient-specific implant design, orthognathic surgery planning, bone reconstruction, and unilateral fracture restoration [35,36]. However, theoretically, no complete symmetry exists in the maxillofacial region [37,38]; therefore, no standard MSP and standard process to establish MSP can be used for the clinical.

In the early days, the way to establish MSP was usually based on 2D imaging of anatomic landmarks [22]. However, the maxillofacial area is a complex 3D structure; furthermore, posture errors may occur when the patient takes images, and reliance on only 2D images cannot accurately reflect the patient’s true maxillofacial structure [39,40]. Research by Berlin et al. [41] proved that the symmetry analysis method based on different lines and anatomic points of the 2D image is inaccurate, and it is impossible to find the best symmetry plane using the data from the 2D image alone. With the popularization of 3D technology, the establishment of symmetry analysis through stereo imaging provides more accurate anatomic indicators for finding the best MSP. Ajmera et al. [42] systematically reviewed the literature for mid-sagittal plane establishment approaches, which are most commonly based on three techniques: cephalometric MSP, morphometric MSP, and a symmetry plane. Cephalometric MSP relies on anatomical landmarks and involves digitizing these landmarks manually or digitally. The morphometric MSP technique is undertaken semi-automatically using land-marks or fully automatically without landmarks. The symmetry plane is a landmark-independent technique for constructing an MSP which can be done semi-automatically or automatically. The cephalometric method has been evidenced to be reliable, highly familiar, and a simple technique which is convenient in the clinical setting [43,44]. Ajmera et al. found cephalometric and morphometric approaches to be equally effective [45]. Despite cephalometric MSP is subjective and time-consuming, it is simple and user-friendly for routine clinical use. The morphometric MSP approach may be quicker to implement, landmark-dependent and more reliable, however the cost of the software and hardware and the need for additional training should not be overlooked. The fully automated approach based on a ‘Symmetry plane’ may not be applicable clinically due to the inaccessibility of the in-house developed software [42].

Technical template mapping and 3D surface-to-surface deviation analysis have become important scientific tools in skeletal morphology [46], which were used in this study to mirror and superimpose a mandibular model onto a 3D color map to precisely identify symmetry. In this study, the three most common MSPs were selected for clinical analysis to evaluate the effectiveness of the symmetry plane in an intuitive way—that is, with the comparison of the original 3D skull model and the mirror model in an overlapped heat map, each point of 3D model was the unit for comparative analysis, allowing the overall overlap precision to be calculated. The visualized color and overlap precision directly reflected the rationality and accuracy of the symmetry plane. The results demonstrated significant statistical differences between the three MSPs (N-ANS-PNS, N-ANS-S, N-Ba-S) and that the plane N-ANS-PNS may be the best choice for the MSP in clinical applications. Likewise, the present review demonstrated that cephalometric MSP based on N-ANS-PNS was consistent with the symmetric mid-sagittal reference plane [43]. For the same MSP, women had better symmetry than men, whereas age, height, and weight had no significant impact on symmetry. In the 3D chromatic deviation mapping, the color results showed that the green area had the largest distribution, reflecting the higher degree of matching of the two models in the frontal bone, anterior zygomatic part, and maxillary and mandible body. Combined with the results of the average threshold difference, it can be concluded that the plane N-ANS-PNS can be used as an ideal MSP for the patient, whereas the N-ANS-S plane is less effective for evaluating the symmetry of the maxillofacial region, and it is more difficult to evaluate the symmetry of the maxillofacial region using N-Ba-S as the MSP. Therefore, in the clinical application of MSP to restore or reconstruct the frontal bone, anterior zygomatic part, and maxillary and mandible body, the plane N-ANS-PNS can be selected as MSP in priority to achieve the expected effect most quickly and accurately.

In this study, the innovative application of mirror technology combined with 3D chromatic deviation mapping effectively evaluated the accuracy of MSP. However, although we used the method of repeated positioning by the same physician to reduce the error of anatomic landmark positioning, error was still unavoidable. Differences between male and female may require further study to determine whether such differences are biological or not. The subjects of this study were all Chinese, and the applicability of the findings to all populations needs to be further explored.The 3D chromatic deviation mapping also proved that plane N-ANS-PNS as MSP was not suitable for repair and reconstruction of temporal bone, condyle, and ascending ramus of mandible. Further research is needed to find more suitable plane as MSP, or to find new ways to repair and reconstruct the above areas.

## 5. Conclusion

In summary, the establishment of the MSP is critical in the evaluation of facial symmetry, and the ideal MSP has important clinical significance. This study supports that the use of different facial midline anatomic landmarks produces differences in the MSP. To treat patients with disease in the frontal bone, anterior zygomatic part, and maxillary and mandible body to achieve a symmetrical face, the N-ANS-PNS plane can be used as a relatively ideal MSP. For this MSP, women had better symmetry than men. Combined with 3D reconstruction and mirroring technology, use of the N-ANS-PNS plane as an ideal MSP will help surgeon more effectively and reliably complete clinical treatment in maxillofacial surgery such as bone reconstruction and orthognathic surgery.
